# Heterogenous electrophysiological features in early stage of hereditary transthyretin amyloidosis neuropathy

**DOI:** 10.1007/s10072-023-07140-w

**Published:** 2023-10-23

**Authors:** Stefano Tozza, Giovanni Palumbo, Daniele Severi, Aniello Iovino, Emanuele Spina, Francesco Aruta, Emanuele Cassano, Rosa Iodice, Raffaele Dubbioso, Lucia Ruggiero, Maria Nolano, Lucio Santoro, Fiore Manganelli

**Affiliations:** Department of Neurosciences, Reproductive and Odontostomatological Sciences, University of Naples “Federico II”, Via Sergio Pansini, 5, 80131 Naples, Italy

**Keywords:** Amyloidosis, TTR-related neuropathy, Heterogeneous neuropathy, Early-stage disease

## Abstract

**Introduction:**

Hereditary transthyretin-mediated amyloidosis (ATTRv, v for variant) is a progressive disease caused by mutations in the *TTR* gene, leading to sensory-motor, axonal and length-dependent neuropathy. However, some patients may show variable electrophysiological pattern. The aim of this study was to evaluate the electrophysiological features of TTR amyloid neuropathy at the time of the first nerve conduction study (NCS) to assess whether there were distinguishing features useful for early diagnosis.

**Methods:**

We retrospectively revised the first electrophysiological findings of ATTRv patients, and we categorized the neuropathy based on nerve conduction slowing, type of involved fibres and distribution pattern of PNS involvement. Cluster analysis was performed to evaluate the prevalence of neuropathy features between the early and late stage of disease, based on disease duration and disability burden assessed by NIS.

**Results:**

We recruited 33 patients (27 males) with mean age 63.9 ± 10.8 years, mean disease duration 2.8 ± 2.4 years and mean NIS 47.6 ± 41.8. Overall, the frequency analysis showed that the most common features of ATTRv neuropathy included the categories of axonal, sensory-motor and neuronopathic-like pattern. This electrophysiological pattern of PNS involvement was constant in patients in late stage of disease, whereas ATTRv patients in early stage of disease displayed variable electrophysiological pattern of PNS involvement.

**Discussion:**

Our findings demonstrated that ATTRv neuropathy may present at first NCS in a variable way, and it changes over the course of disease. Such heterogeneity makes the suspicion of ATTRv even more challenging at the time of first electrophysiological examination.

## Introduction

Hereditary transthyretin amyloidosis (ATTRv, v for variant) is a multisystemic disorder caused by mutation in the *transthyretin* (*TTR*) gene with a progressive involvement of peripheral nervous system (PNS). The mutated TTR tetramer dissociates in monomers that misfold and aggregate in amyloid fibrils accumulating in PNS [1]. In detail, amyloid fibril deposit starts in the dorsal root ganglia (DRG) and autonomic ganglia, causing an early axonal loss of sensory nerve fibres through a dying-back degeneration. Subsequently, TTR deposition may involve nerve roots and peripheral nerves leading to a progressive sensory-motor axonal neuropathy [2].

Accordingly, the neuropathy in ATTRv was labelled as a progressive, length-dependent, sensory or sensory-motor axonal neuropathy. However, ATTRv diagnosis is challenging with high rate of misdiagnosis, inappropriate treatment and diagnostic delay [3]. In the era of different disease modifying treatments, a proper and early diagnosis is essential to limit the disability burden and assure the halting of disease progression [4].

The aim of this study was to evaluate the electrophysiological features of neuropathy in 33 ATTRv patients at the time of the first neurophysiological evaluation and to assess whether there were distinguishing features useful for early diagnosis.

## Methods

This is a monocentric and retrospective study that re-evaluated the first neurophysiological evaluation of ATTRv patients. We included patients carrying a pathogenic mutation in TTR gene that underwent nerve conduction study (NCS) at our third level center from 2011 to 2023. Patients with incomplete clinical and electrophysiological data were excluded from the analysis.

Electrophysiological data was re-evaluated from internal database and was collected to establish the features of neuropathy based on nerve conduction slowing, type of involved fibres and distribution pattern of PNS involvement.

In detail, in the “nerve conduction slowing” feature, we included three categories: (1) demyelinating neuropathy: motor (MNCV) and/or sensory nerve conduction velocities (SNCV) met the EAN/PNS criteria of demyelination [5], (2) axonal neuropathy: reduction of compound action potential (CMAP)/sensory action potential (SAP) amplitude and MNCV/SNCV normal or slightly slowed but not fulfilling demyelinating criteria [5] and (3) intermediate neuropathy: normal CMAP/SAP amplitude and MNCV/SNCV reduced but not fulfilling demyelinating criteria [5].

In the “type of involved fibres” feature, we included three categories: (1) sensory predominant neuropathy: SAPs absent or reduced ≥ 30% of lower limit normal value (LLN) with normal o slightly reduced CMAPs, (2) motor predominant neuropathy: CMAPs absent or reduced ≥ 30% of LLN with normal o slightly reduced SAPs and (3) sensory-motor neuropathy: SAPs and CMAPs similarly impaired.

In the “distribution pattern” feature, we included four categories: (1) length-dependent neuropathy: lower limb nerves more impaired than upper limb nerves, (2) upper limb predominant neuropathy: upper limb nerves more impaired than lower limb nerves, (3) neuronopathic-like: upper and lower limb nerves equally impaired and (4) multi-neuropathy: side-to-side nerve CMAP/SAP asymmetry (≥ 50%) or inhomogeneous involvement of two contiguous nerves (not explained by entrapment neuropathy).

Lastly, gender, mutation, age at first nerve conduction study (NCS), disease duration, familial amyloid polyneuropathy (FAP) stage and Neuropathy Impairment Score (NIS) were recorded in a minimal clinical dataset.

### Statistical analysis

Descriptive analyses, based on the mean ± standard deviation (SD) for numerical variables and percentage for categorical data, were used to define the prevalence of the most frequent features of ATTRv neuropathy.

In order to evaluate whether the neuropathy features may change over time, we used K-mean cluster analysis [6] to divide our cohort in two clusters—the cluster 1 (early stage) and cluster 2 (late stage). We set up the criteria to recognize early vs late-onset cluster in our cohort based on disease duration and disability burden assessed by NIS. We consequently analyzed the prevalence of neuropathy features of each cluster.

Analyses were performed using the statistical software IBM SPSS Statistic version 25.

## Results

We included 33 ATTRv patients (M/F = 27/6) belonging to 25 families. Fourteen patients carried V30M variant, 15 F64L, 3 E54L and 1 V122I. Twenty of them were in FAP stage 1, 8 in FAP stage 2 and 5 in FAP stage 3. Patients presented at first NCS a mean age of 63.9 ± 10.8 years, a mean disease duration of 2.8 ± 2.4 years and a disability burden assessed by NIS of 47.6 ± 41.8 points (Table 1).

**Table 1:** Clinical and electrophysiological findings

Age at first NCS (years)	63.9 ± 10.8 (31–76)
Disease duration (years)	2.8 ± 2.4 (0–10)
Mutation	
V30M F64L E54L V122I	14 (42.4%) 15 (45.6%) 3 (9%) 1 (3%)
FAP stage	
FAP 1 FAP 2 FAP 3	20 (60.6%) 8 (24.2%) 5 (15.2%)
NIS	47.6 ± 41.8 (0–147)
Nerve conduction slowing	
Axonal Demyelinating Intermediate	29 (87.8%) 2 (6.1%) 2 (6.1%)
Type of involved fibres	
Sensory Motor Sensory-motor	6 (18.1%) 2 (6.1%) 25 (75.8%)
Distribution pattern	
Length-dependent Upper limb predominant Neuronopathic-like Multiplex neuropathy	10 (30.3%) 3 (9.1%) 16 (48.5%) 4 (12.1%)

*NCS* nerve conduction study, *FAP* familial amyloid polyneuropathy, *NIS* Neuropathy Impairment Score

The analysis of NCS features revealed that the most frequent “nerve conduction slowing” category was axonal (87.8% vs demyelinating 6.1%; intermediate 6.1%), the most frequent “type of involved fibres” category was sensory-motor (75.8% vs sensory 18.1%; motor 6.1%) and the most frequent “distribution pattern” category was neuronopathic-like (48.5% vs length-dependent 30.3%; upper limb 9.1%; multi-neuropathy 12.1%) (Table 1).

**Fig. 1:**
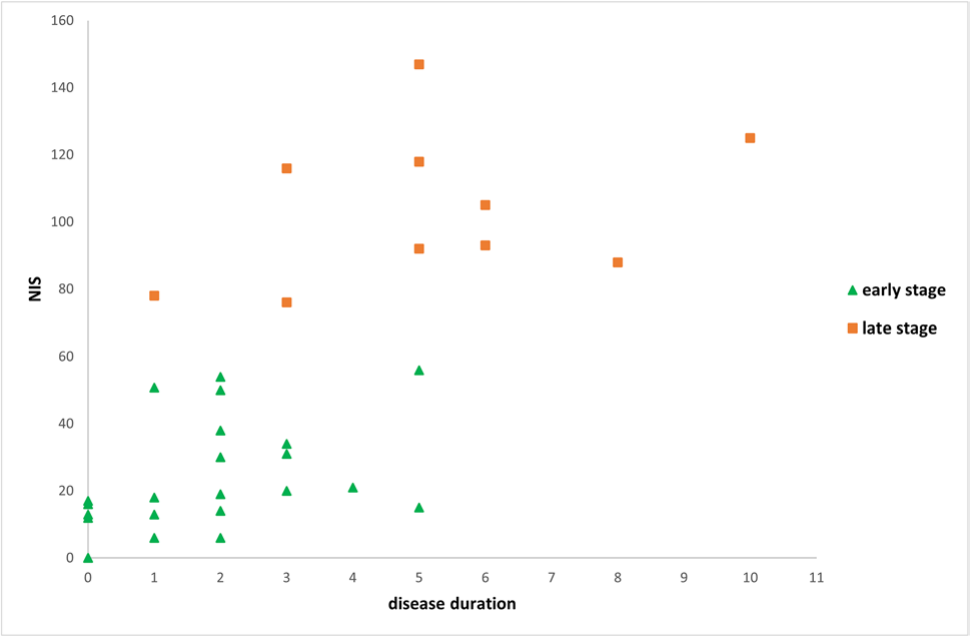
Early and late stage clusters. Figure showed early and late stage clusters divided according to disease duration and disability burden (assessed through the NIS). NIS, Neuropathy Impairment Score

The K-mean analysis showed the presence in our cohort of the two distinct clusters (*p* < 0.001): in the early-stage patients had a disease duration of 1.8 ± 1.5 years and NIS of 23.2 ± 16.8 points, while the late-stage cluster showed a disease duration of 5.2 ± 2.5 years and disability burden of 103.8 ± 22.6 points (Fig. 1). The frequency analysis showed that neuropathy features were different between the two clusters. In the late-stage cluster, the neuropathy was exclusively axonal, sensory-motor and with neuronopathic-like pattern, whereas in the early stage, neuropathy features were heterogenous, including intermediate NCV, sensory and/or motor predominant nerve fibre involvement and variable distribution pattern (length-dependent, upper limb predominant, multi-neuropathy) (Table 2).

**Table 2:** Prevalence of electrophysiological findings between the early and late stage cluster

	Cluster 1 (early stage)	Cluster 2 (late stage)
Number of patients	23	10
Age at first NCS	61.9 ± 12.3 (31–76)	68.6 ± 3.5 (64–75)
Disease duration	1.8 ± 1.5 (0–5)	5.2 ± 2.5 (1–10)
NIS	23.2 ± 16.8 (0–56)	103.8 ± 22.6 (76–147)
Nerve conduction slowing		
Axonal Demyelinating Intermediate	20 (86.9%) 1 (4.4%) 2 (8.7%)	9 (90%) 1 (10%) 0 (0%)
Type of involved fibres		
Sensory Motor Sensory-motor	6 (26.1%) 2 (8.7%) 15 (65.2%)	0 (0%) 0 (0%) 10 (100%)
Distribution pattern		
Length-dependent Upper limb Neuronopathic Multiplex neuropathy	10 (43.5%) 3 (13.1%) 6 (26.1%) 4 (17.3%)	0 (0%) 0 (0%) 10 (100%) 0 (0%)

*NCS* nerve conduction study, *NIS* Neuropathy Impairment Score

## Discussion

ATTRv neuropathy is a rare, multisystemic and fatal disease. In the last decade, several disease modifying treatments have become available, and they are more effective in slowing or halting disease progression, the earlier they are administrated [7–9]. Therefore, early diagnosis is crucial for limiting disability burden.

However, the diagnosis of ATTRv neuropathy is still delayed and patients may experience misdiagnosis and sometimes inappropriate therapy as well [10]. The aim of this study was to define the heterogeneity of ATTRv neuropathy at first NCS.

Overall, the frequency analysis showed that the most common features of ATTRv neuropathy included the categories of axonal, sensory-motor and neuronopathic-like pattern.

The neuropathy was classifiable as primary axonal in most patients, and noteworthy, CMAP/SAP amplitude was constantly reduced in patients displaying demyelinating features as well [11], suggesting that a severe axonal loss in patient with a diagnosis of CIDP with a progressive course and a poor response to immunomodulatory therapy should raise the suspicion for ATTRv neuropathy. Indeed, demyelination may occur in ATTRv patients as amyloid deposit may cause myelin sheet detachment and nodal enlargement [12]. Interestingly, 6% of our patients displayed slightly reduced NCV not fulfilling EAN/PNS criteria [5] and normal CMAP amplitude (intermediate category). In such patients, we might speculate that TTR oligomers interact with ion channels and, hence, alter the homeostasis of the nerve membrane and impair NCV before nerve damage has occurred [13, 14].

The most frequent “type of involved fibres” category was sensory-motor neuropathy, while sensory or motor predominant neuropathy appeared rarer in our cohort. If sensory predominant neuropathy can be explained by early involvement of DRG, for predominant motor neuropathy, the pathomechanism is more difficult to figure out. Anyway, TTR-related predominant motor neuropathy should not be overlooked as it can be also misdiagnosed as lower motor neuron disease [10, 15].

Lastly, 50% of patients had a neuropathy with neuronopathic-like pattern. Such findings fit with the notion that nerve damage starts proximally (DRG and nerve roots) and, accordingly, the involvement of upper limb nerves can be more precocious than in length-dependent neuropathy [16]. In keeping with this, skin biopsy demonstrated an early and highly frequent of peripheral nerve involvement with a non-length dependent fashion (ganglionopathy) in ATTRv patients and carriers [17, 18]. Moreover, the rapidly progressive feature of ATTRv neuropathy means that even a neuropathy starting as length-dependent may soon involve the upper limbs, thus, resulting in a neuronopathic-like pattern.

Interestingly, we also observed upper limb predominant neuropathy in 10% and multi-neuropathy in 12% of our cohort of patients. Upper limb onset (ULO) of ATTRv may be quite common in non-endemic areas [19], and the pathomechanism underling the nerve damage seems to be different from other form of ATTRv neuropathy. Pathological data from radial nerve biopsy showed that ULO patients had a significant loss of large myelinated fibres with conspicuous amyloid deposits in endoneurium as well as in small epineurial vessels walls, leading to vessel obstruction and eventually to chronic ischaemia due to amyloid angiopathy [19].

On the other hand, multi-neuropathic involvement of PNS has been only anecdotally reported and such involvement could reflect a random heterogeneous deposition of amyloid along the nerve [20].

The cluster analysis showed that ATTRv patients in late stage of disease (longer disease duration and higher disability) had a constant electrophysiological pattern of PNS involvement consistent with an axonal, sensory-motor and neuronopathic-like neuropathy. Conversely, ATTRv patients in early stage of disease (shorter disease duration and lower disability) displayed variable electrophysiological pattern of PNS involvement for “nerve conduction slowing”, “type of involved fibres” and “distribution pattern”.

In conclusion, although the limits of the study (retrospective design and relatively small sample), our findings demonstrated why ATTRv diagnosis is so challenging since the neuropathy may present at first NCS in variable way and it changes over the course of disease. If in early stage of the disease, neuropathy can be extremely heterogeneous, it can rapidly evolve in a severe polyneuropathy characterized by a sensory-motor axonal involvement with a neuronopathic-like pattern.

Generally, in clinical practice, patients with progressive axonal and sensory-motor neuropathy are addressed to genetic analysis if at least one red flag is detected (e.g., carpal tunnel syndrome, lumbar stenosis, cardiac, ocular, renal involvement, and autonomic symptoms). However, clinicians should not overlook the hypothesis of ATTRv neuropathy when facing with different electrophysiological features. Clinicians should perform the *TTR* genetic analysis in patients with neuronopathic-like polyneuropathy, while in patients with other type of polyneuropathy, they have to look for ATTRv multisystemic involvement (red flags) in order to suspect ATTRv disease and thus perform an early diagnosis.

Lastly, such heterogeneity at the time of presentation should be carefully considered when investigating presymptomatic subjects, as if an extensive NCS is not performed (bilaterally upper and lower limb nerves), PNS involvement may be missed, resulting in a delay of treatment.
